# Revealing the Role of Microstructure and Strain Heterogeneities in the Elastic–Plastic Transition of Glassy Polymers

**DOI:** 10.1021/acs.macromol.5c00694

**Published:** 2025-07-03

**Authors:** Hilal Reda, Panayiota Katsamba, Anthony Chazirakis, Vagelis Harmandaris

**Affiliations:** † Computation-based Science and Technology Research Center, 338376 The Cyprus Institute, Nicosia 2121, Cyprus; ‡ Institute of Applied and Computational Mathematics, Foundation for Research and Technology - Hellas, Heraklion GR-71110, Greece; § Department of Mathematics and Applied Mathematics, 37777 University of Crete, Heraklion GR-71110, Greece

## Abstract

In this work, we investigate the atomic and microstructural underpinnings of glassy polymers, focusing on the transition from elastic to plastic regimes under tensile strain and the underlying mechanisms at the atomic scale via detailed atomistic simulations. We highlight the role of local heterogeneities and their interplay in stress, strain, and density fields. Our key message is that the coupling between the microstructure (density) and strain heterogeneities is crucial for the elastic–plastic transition. Regions with increased free volume facilitate activation of vitreous segments, reorganization of polymer atoms to minimize nonbonded interactions and stress dissipation, leading to enhanced mobility and delayed strain-hardening of low-density regions.

## Graphical abstract

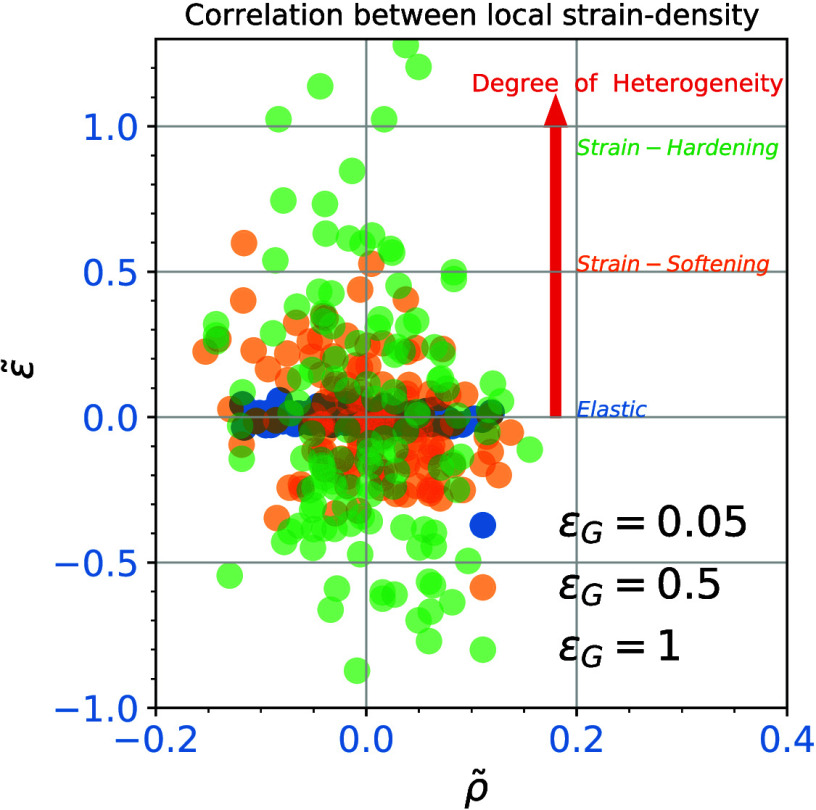

## 1 Introduction

Due to their fundamental and technological importance, the mechanical and dynamical properties of polymer glasses have been intensively studied over the past few decades via experimental techniques^1–6^ but also through molecular dynamics simulations mainly in the linear elastic regime.^7–13^ The elastic–plastic transition in glassy polymers is, in particular, of crucial importance in understanding their mechanical behavior, and thus, it has been extensively studied using several experimental techniques, including, among others, mechanical tests,^14–17^ NMR,^18^ positron annihilation lifetime spectroscopy,^19^ and dielectric spectroscopy.^18,20^ These experiments indicate that the segmental mobility can be considerably higher during external deformation beyond the yield point. Moreover, the contribution of the entanglement network and segmental motion to the total response to strain hardening of a number of linear and cross-linked polymer glasses was experimentally studied, suggesting an empirical modification of the so-called compressible Leonov model by a strain-dependent activation volume.^21^

In addition to experimental work, a large body of theoretical and simulation research has been carried out to investigate the molecular mechanisms responsible for yielding during deformation and to understand the plasticity phenomena of glassy polymers.^22^ Most theoretical works share the common idea that stress would lower the potential barrier and that plastic regimes can be reached through the activation of vitreous segments, thus allowing a more efficient reorganization of polymer segments. It is well-known that the yield occurs when the segmental (α) relaxation time becomes comparable to the experimental time scale, that is, the reciprocal of the deformation rate. The Eyring-like idea of activation attempts to explain how plasticity emerges but does not clarify when this mechanism ceases to work and does not concern the complex and evolving mechanisms that govern deformation beyond the yield point.^23^ Recently, Schweizer and co-workers developed a comprehensive force-level microscopic theory based on the nonlinear Langevin equation (NLE), an approach conceptually similar to that of the energy landscape. The authors highlighted the amplitude of the density fluctuation as the key variable that affects the segmental transient localization and relaxation processes. This mean-field theory provides a microscale-based approach for understanding how individual polymer segments behave under deformation, connecting molecular-level behavior to macroscopic mechanical properties.^24–26^ Moreover, the elastic–plastic transition of glassy polymers has been investigated by molecular dynamics (MD) simulations, mostly via generic bead-spring coarse-grained models. Robbins et al., in a series of papers, provided new insights on mechanisms related to strain hardening, focusing on large-scale chain relaxation without invoking entanglements.^27–30^ MD simulations, using atomistic or bead–spring models, also report rapid segmental dynamical motions^31–35^ during mechanical plastic deformation that have elevated the potential energy landscape.^35–38^ In addition, the role of chain reorientation in the hardening behavior of glassy polymers has been discussed, mainly by employing the Lee-Kröner decomposition method.^39,40^

The aforementioned works provide very important insights into the elastic-to-plastic transition and strain hardening in glassy polymers for length scales of about one Kuhn segment (the typical size of a bead in standard bead-spring models) and above. However, information on the atomic scale concerning the role of chemical specificity in the nonlinear mechanical behavior of glassy polymers is rather scarce. At the same time, most of the aforementioned works do not consider the role of density modifications and strain heterogeneities within the amorphous glassy systems during deformation.

In this work, we provide a detailed investigation, at the atomic scale, of spatial local heterogeneities in microstructure (local packing and density variations) and strain at the onset of plastic deformation, focusing on how atomic interactions influence the mechanical response during deformation of glassy polymers. As a model example, we consider poly­(ethylene oxide), PEO, glassy model systems.

The primary goal of our work is to provide a fundamental investigation of the elastic–plastic transition in amorphous polymers by examining the role of local microstructural heterogeneities, including variations in local packing density, strain–stress distribution, and atomic scale interactions. Through MD simulations, we aim to uncover how these factors influence the mechanical response of poly­(ethylene oxide) during deformation. The manuscript is structured as follows: Section 2 details the materials and methods, including the preparation of the atomistic structure, the deformation setup, and simulation parameters. In Section 3, we present and discuss our key findings. We first analyze the overall stress–strain behavior of glassy polymeric systems, followed by an investigation of polymer chain reorientation during deformation. Then, we examine the spatial distribution of the local strain field and explore the coupling between local density fluctuations and strain heterogeneities. Finally, Section 4 summarizes our conclusions and provides perspectives on future research directions.

## 2 Methods, Model, and Simulation Details

### 2.1 Model and Atomistic Simulations of the PEO Systems

The initial step of the methodology is to obtain several (here, ten) initial uncorrelated configurations of the amorphous glassy poly­(ethylene oxide), PEO, system. First, long atomistic molecular dynamics simulations were performed for a homogeneous bulk PEO system at a high temperature. The polymer matrix consisted of 48 PEO chains terminated with methyl groups. The PEO chains are monodisperse, each consisting of 50 monomers, with a molecular weight that about the entanglement molecular weight Me of linear PEO (Me = 2020 g/mol), which corresponds to about Ne = 46.^41^ The entanglement length of the glassy PEO was determined using the Z1 code.^42^ Then, a well-equilibrated PEO melt configuration was cooled to a temperature well below *T*_g_ at 150 K to obtain a glassy PEO system. The above procedure was repeated for several uncorrelated PEO configurations. Polymer chains were modeled using a modified TraPPE force field.^43,44^ Electrostatic interactions were evaluated using the particle mesh Ewald (PME) method.^45^ Simulation runs were performed in the NPT ensemble, where the pressure was kept close to 1 bar with the Parrinello–Rahman barostat^46^ while the temperature was maintained constant using the Berendsen thermostat.^47^

After preparing the glassy samples, we applied uniaxial deformation with a constant strain rate *ε̇* = 10^–5^ fs^–1^. The effect of strain rate on the mechanism of polymer nanocomposite reinforcement deep in the glassy state can be found in our previous work.^12,48^

A snapshot of an arbitrary chain during deformation is given in Figure 1. The deformation was performed using LAMMPS with a Nose–Hoover thermostat and barostat. More information on the preparation of the systems and the deformation process can be found in previous work.^13^

**Figure 1:**
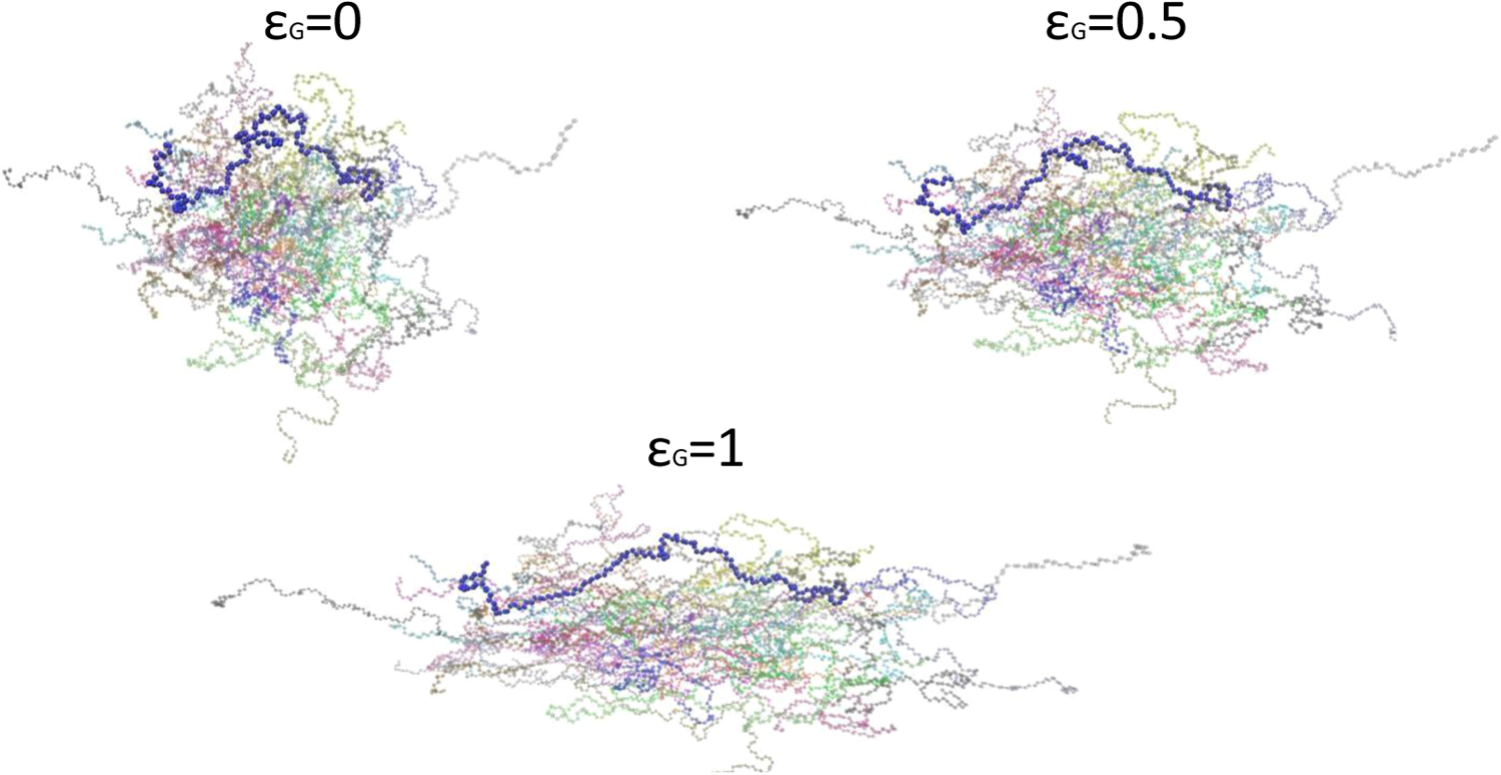
Snapshots of the atomistic PEO model system with highlighting an arbitrary polymer chain during deformation. Three different values of global strain are presented at: equilibrium (ϵ_G_ = 0), strain-softening (ϵ_G_ = 0.5), and strain-hardening regimes (ϵ_G_ = 1).

### 2.2 Analysis Methods

In order to compare the simulation results with theoretical predictions, we use the Weibull model that is widely adopted for the failure mechanism, fracture statistics, and strength distribution of glassy systems.^49,50^ The Weibull model is modified here to characterize the stress induced in the deformation process in the elastic and strain-softening regime, similarly to the work in ref 51. In this modified formulation, a linear stress–strain relationship is accounted for by a nonlinear exponential function, $\sigma_{\mathrm{wel}}=E_{\mathrm{wel}}\epsilon \times \exp\left(-{\left(\frac{\epsilon }{\epsilon_{0}}\right)}^{m}\right)$ , where *E*_wel_ is called the Weibull modulus, ϵ_0_ is a characteristic strain, and *m* is the shape parameter that determines the shape.

Moreover, in the strain-hardening regime, we compare the simulation results with the Gaussian strain-hardening model^10,28,52^ describing the stress–strain relationship in the strain-hardening regime as σ = σ_0_ + *G*_R_g­(λ), where *G*_R_ is the hardening modulus, $g\left(\lambda \right)=\frac{1}{\lambda }-\lambda^{2}$ provides the functional form of the hardening, and the constant offset σ_0_ is added to fit the initial yield stress. In practice, for the adjustment of the stress–strain curve in the elastic and strain-softening regimes, that is, from the strain values 0 to 0.5, the adjustment parameters are determined as ϵ_0_ = 0.11—corresponding to the maximum strain of the stress–strain curves, the shape parameter *m* as 0.64, and the Weibull modulus *E*_wel_ at approximately 6.64 *GPa*.

To describe the reorganization of the PEO glassy systems during deformation, at the atomic scale, we consider the autocorrelation vector function *P*_2_, defined as

1 $$P_{2}\left(\varepsilon \right)=\frac{1}{2}\sum_{I=1}^{N_{b}}\left[3\cos^{2}\theta_{I}^{b}\left(\varepsilon \right)-1\right]$$

with *N*_b_ being the total number of bonds and θ_I_^b^(*ε*) being the angle formed between the bond vector I at deformation *ε* and the same vector at equilibrium (*ε* = 0). Furthermore, we investigate the alignment of polymer chains with respect to the deformation axis by considering a vector orientation function *P*_2_^*xx*^ (order parameter), but in this case, θ_I_^b^(*ε*) being the angle formed between the bond vector I at deformation *ε* and the axis of deformation *x*.

A characteristic critical value of the deformation *ε*, with respect to the reorientation of chain segments, can be extracted by fitting *P*_2_(ϵ) to the stretched exponential Kohlrausch–Watts equation: $P_{2}\left(\varepsilon \right)=P_{0}\exp{\left(-\frac{\epsilon }{\epsilon_{c}}\right)}^{\beta }$ , where *P*_0_, ϵ_c_, and β are the fitting parameters, and ϵ_c_ is reported as the critical deformation. Another key feature used to study the transition is the volumetric strain given by ϵ*_v_* = ϵ_*x*_ + ϵ_*y*_ + ϵ_*z*_.

To investigate the spatial distribution of the (local) mechanical properties of the model polymer systems, the stress, strain, and deformation gradient at the atomic level resolution are calculated as in refs 53,54 . We thus compute the value of the stress per atom (local stress) as given by the Virial formalism. Concerning the local strain and the deformation gradient, here we use a recently proposed methodology to directly probe the strain field and the deformation gradient in the model polymer at the atomic level.^53^ At the initial stage, the deformation gradient **F** ^α^ and the strain order deformation gradient **G** ^α^ for each atom α are calculated through a minimization problem related to the position of the atom of interest in its neighboring atoms within the cutoff radius *r*_cut_,

2 $$\left\{F^{\alpha },G^{\alpha }\right\}=\underset{F^{\alpha }\;,G^{\alpha }\;}{\mathrm{argmin}}\sum_{\beta =1}^{N}{∥\Delta x^{\alpha \beta }-F^{\alpha }\left(x\right)\cdot \Delta X^{\alpha \beta }-\frac{1}{2}G^{\alpha }:\left(\Delta X^{\alpha \beta }⊗\Delta X^{\alpha \beta }\right)∥}^{2}$$

where *N* is the number of neighboring atoms of α, **x** ^α^ is the position of atom α in the current configuration Ω_,_, and **X** ^α^ is the position in the reference configuration Ω_0_. When **F** ^α^ and **G** ^α^ are determined in this manner, the Lagrangian Green strain tensor **E** ^α^ and the strain gradient tensor **K** ^α^ are defined with respect to reference coordinates as

3a $$E^{\alpha }=\frac{1}{2}\left(F^{\alpha }\cdot {\left(F^{\alpha }\right)}^{T}-I\right)$$

3b $$K^{\alpha }=\left[{\left(F^{\alpha }\right)}^{T}\cdot G^{\alpha }\right)]$$

The computation of **E** ^α^ and **K** ^α^, with respect to the reference coordinates, allows us to probe the distribution of the (local) strain fields, as well as the strain gradient on the atomic scale. The gradient of the deformation tensor is an extension of the classical strain tensor represented by the Cauchy constitutive law, incorporating higher-order spatial derivatives of displacement to account for strain gradients. This approach is particularly relevant in the study of nanostructured materials, where traditional continuum mechanics cannot accurately describe the microstructure effect on the atomic/molecular scale. For more details about the calculation of the local strain, please refer to ref 55. As an example, the formation of voids at the nanoscale and the resulting increase in the local heterogeneities within the plastic region significantly influence the deformation behavior of the materials. In such cases, the deformation gradient can be a critical parameter in describing the strain localization phenomenon. More information on the computation of the local strain can be found in our previous work.^12,53^

## 3 Results and Discussion

### 3.1 Overall Stress–Strain Behavior of Glassy Polymeric Systems

We start with the investigation of the model PEO systems by probing their overall (global) mechanical behavior. In Figure 2a, we present the engineering stress–strain diagram (shown in green) and the various regimes therein. Stress is always computed as a difference with respect to the equilibrium values of a specific configuration. Note also that we denote ϵ_G_ as the applied global strain and ϵ as the local one. At low strains below ϵ_G_ < 0.1, the stress increases almost linearly with a gradient of about 3 GPa (Young’s modulus), as the material resides in the elastic (Hookean behavior) regime. Stress increases until it reaches its maximum value of σ_*Y*_ = 0.26 GPa at ϵ_G_ = 0.14, after which there is a significant decrease in stress. The peak of the stress–strain curve is intimately connected to the transition to the plastic regime, in which the imposed strain causes permanent deformation of the material. The region after the peak is referred to as strain softening of the material due to the substantially reduced stress response of the material at these larger strains. After the strain-softening regime (ϵ_S_ = 0.6), strain hardening is observed as a progressive strengthening of a material during plastic deformation that is usually associated with ductility, a property often sought after in polymer materials to postpone fractures and failure.

**Figure 2:**
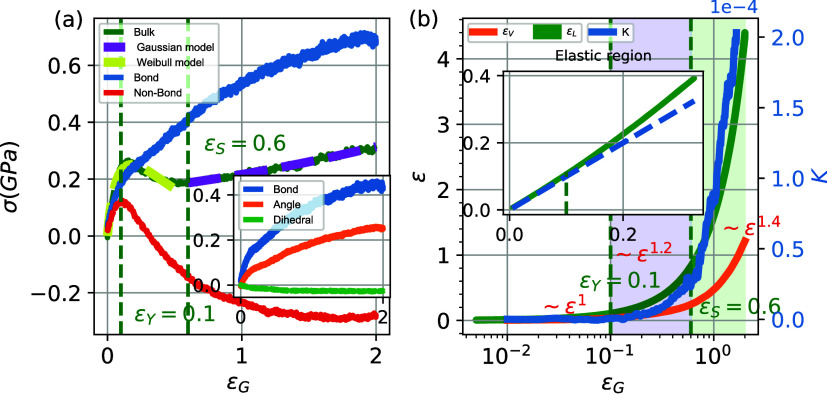
(a) Stress–strain diagram of glassy PEO and the nonbonded and bonded interactions as a function of the imposed global strain, ϵ_G_. (Inset) Contribution of bond, angle, and dihedral interactions in stress. (b) Evolution of average local strain ϵ_L_ and volumetric strain ϵ_V_ (left axis) and gradient of deformation (right axis). (Inset) Evolution of the average local strain in the elastic region. The dashed blue line represents the global deformation ϵ_G_ applied to the system.

It is of interest to examine how constitutive models describe the simulation results. For this, in Figure 2, we compare the stress–strain behavior of the glassy PEO systems with the predictions of standard semiempirical constitutive models in the linear-plastic and strain-hardening regimes. We note the excellent agreement of the stress–strain curve (green-dashed line in Figure 2a) with a modified version of the Weibull distribution model, $\sigma_{\mathrm{wel}}=E_{\mathrm{wel}}\epsilon \times \exp\left(-{\left(\frac{\epsilon }{\epsilon_{0}}\right)}^{m}\right)$ , where *E*_wel_ is called the Weibull modulus, ϵ_0_ is a characteristic strain, and *m* is the shape parameter that determines the shape for the elastic and strain-softening regimes (yellow dashed line). For higher values of the deformation in the strain-hardening regime, the predictions of the Gaussian strain-hardening model, σ = σ_0_ + *G*_R_g­(λ), where *G*_R_ is the hardening modulus and $g\left(\lambda \right)=\frac{1}{\lambda }-\lambda^{2}$ provides the functional form of the hardening, which are in good agreement with the simulation data. The constant offset σ_0_ is added to fit the initial yield stress, and λ is the stretching for the strain-hardening region (magenta dashed line). The Weibull model describes the linear regime well using ϵ_0_ = 0.11—corresponding to the maximum strain of the stress–strain curves, the shape parameter *m* as 0.64, and the Weibull modulus *E*_wel_ at approximately 6.64 GPa. The Gaussian strain model fits the strain-hardening regime very well, as we can see in Figure 2a where σ_0_ = 0.15 GPa and the strain-hardening parameter *G*_R_ = 20 MPa, and these values are close to the range of values reported in the literature for other glassy polymers, such as polycarbonate (PC) and polystyrene (PS).^52^

Next, we investigate the role of different energetic contributions (bonded and nonbonded) in the mechanical response of the glassy polymer by presenting, in Figure 2a, the decomposition of the Virial part of the stress into the bonded (bond, angle, and dihedral) and nonbonded interactions. This methodology closely follows the decomposition approach described by Tang et al.^56^ The bonded stress increases monotonically, indicating the absorption of the strain energy into the stretching of the chemical bonds and bond angles, as expected. The nonbonded stress increases nonlinearly in the elastic region, reaching its maximum value at a strain value slightly lower than the maximum of the total stress, which coincides with the critical yield strain value of ϵ_Y_ = 0.1. Beyond this strain, the nonbonded stress decreases, and this gives rise to the plastic behavior of the material (strain-softening regime), despite the increasing bonded stress. This is a clear indication of the reorganization of polymer segments (activation of vitreous segments) during deformation to reduce the number of energetic interactions. Interestingly enough, such an activation occurs almost entirely via nonbonded interactions, and only partially via the decrease of the dihedral interactions. Beyond ϵ_s_ = 0.6, the nonbonded stress converges to a plateau-like value, while the bonded stress continues to increase, leading the material to enter the strain-hardening regime. We also note the different behaviors of increasing bonded stress in the elastic, strain-softening, and strain-hardening regimes, with the gradient of bonded stress being further reduced from one region to the other. Upon decomposing the total contribution of bonded energy to the bond, angle, and dihedral components, shown in the inset of Figure 2a, it is clear that bond stretching has the most significant impact on bonded energy, with the bond angle interactions following a similar profile, albeit smaller. We note a very low contribution of dihedral angles (representing the rotational freedom around the bond).

To further investigate the origins of nonlinearity leading to the elastic-to-plastic transition, we probe properties related to the heterogeneities in the local strain microenvironment of the material by computing local strain fields. In Figure 2b, we present data on the average local deformation in the material, ϵ, and the magnitude of the gradient of deformation, *K*, which serves as an indicator of strain localization during deformation.

As is clear from the inset of Figure 2b in the elastic region (ϵ_G_ < ϵ_Y_ = 0.1), the average local deformation matches the global deformation almost perfectly, indicating that the deformation of the material is almost affine. In addition, in the plastic regime, the value of the gradient of deformation is very close to zero, indicating an almost uniform distribution of the local strain field, while the volumetric strain increases proportionally to the global strain. We highlight the emergence of a critical strain ϵ_Y_ = 0.1 (Figure 2b, inset) beyond which the average local strain departs from this linear behavior, and we associate this with the exit of the material from the low-strain linear elastic regime and the entry into the strain-softening regime, with low-strain localization compared to the strain-hardening regime, as indicated by the lower value of K. This critical point emerges as a generic marker of the elastic-to-plastic transition, representing the yield strain, and is connected with the breakdown of the compatible deformation condition (under external deformation, there are no gaps (voids) or overlaps created within the material). Another notable deviation is observed at ϵ_S_ = 0.6, after which the average local strain, volumetric strain, and magnitude of the deformation gradient increase dramatically. This second critical strain matches very well the end of the strain-softening region, at the same value of ϵ_S_ = 0.6, and is an indication of the entry of the material into the strain-hardening regime. It is interesting that the strain-hardening regime manifests itself with high-strain localization and a dramatic increase in volumetric strain. The deviations of strain between different regions are clearly observed from the power law exponent, which exhibits differences within the three different regions of the strain curve (∼ϵ^1^, ∼ϵ^1.2^, ∼ϵ^1.4^). These differences are often indicative of the underlying nonaffine deformation mechanisms and increased local heterogeneities.

### 3.2 Reorientation of Polymer Chains during Deformation

Previous works in the literature have reported rapid segmental dynamical motions as the material leaves the elastic regime toward plastic deformation.^31–35^ Here, to investigate such phenomena, we compute the autocorrelation vector function, as a function of applied deformation ϵ_G_, *P*_2_(ε_G_), for various vectors defined by segments of different lengths along a polymer chain. A faster decay of *P*_2_(ε_G_) indicates faster bond-reorientation, higher mobility of the monomers, and increased dissipation of energy. Data of *P*_2_(ε_G_) are presented in Figure 3a for several polymer segments that are defined as multiples of Kuhn length, *l*_k_. Note that for PEO systems *l*_k_ ≈ 1 nm, corresponding to approximately 2–3 monomers. Interestingly, for all segments investigated, the chain segments exhibit important reorientation phenomena, their onset occurring at a critical strain of ϵ_Y_ = 0.1, which fits well the other markers of the elastic-to-plastic transition encountered so far.

**Figure 3:**
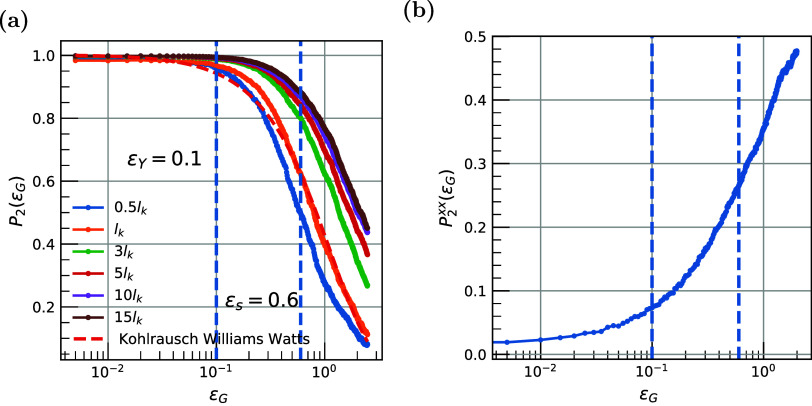
(a) Orientational dynamics of chain segments defined along a polymer chain *P*_2_(ε_G_), as a function of the applied deformation ϵ_G_. Various vectors with length multiples of the Kuhn length *l*_*k*_, which is around 1 nm for PEO,^57^ are shown. Fit with the Kohlrausch–Watts model for 1 *l*_*k*_. (b) Chain orientation “(order parameter)” *P*_2_^*xx*^ (ϵ_G_) of one Kuhn length *l*_*k*_ (which is around 1 nm) with respect to the axis of deformation as a function of the applied deformation ϵ_G_.

Another transition is observed close to the strain values of ϵ_S_ = 0.6, in accordance with the transition from softening to hardening regimes. This transition is observed for 2-mer segments (i.e., approximately one Kuhn length of the PEO chains). The significant reorientation phenomena seen in the profile of *P*_2_(ε_G_) as a function of the imposed strain follow an exponential decay law, as per the Kohlrausch–Williams–Watts equation, as illustrated for the case of segments of one Kuhn length. The critical deformation ϵ_c_ is extracted by fitting *P*_2_(ε_G_) to the Kohlrausch–Watt equation to be 1.1. Consequently, we can define a characteristic stress-induced “relaxation time” during deformation *t*_r_ = ϵ_c_/ϵ̇ = 0.11 ns. It is of interest that such time scales correspond to segmental relaxation of an equilibrium PEO system at high temperatures (melt state) about 100–150 degrees above *T*_G_.

To further investigate the reorientation of chain segments during deformation, with respect to the direction of the applied tensile deformation (*x*-axis), we probe, in Figure 3b, the evolution of the vector orientation function, with respect to the *x*-axis, *P*_2_^*xx*^, as a function of the applied strain, ϵ_G_, for a vector equal to the Kuhn length of the PEO chain. As expected, *P*_2_^*xx*^ increases with an increase in deformation, showing a continuous orientation of the chain segments toward the direction of the applied deformation. The value of *P*_2_^*xx*^ starts from zero at equilibrium (random orientation with respect to the *x*-axis) and approaches a value of around 0.5 at high deformation (a value of 1.0 denotes the perfect alignment of vectors along the *x*-axis).

### 3.3 Evaluation of Molecular Stiffness via the Debye–Waller Factor

To have a better understanding of the influence of deformation on the mechanical response of a glassy polymer, we calculate the molecular stiffness $\frac{1}{\left\langle u^{2}\right\rangle }$ where ⟨*u* ^2^⟩ is the Debye–Waller factor^58^ given by

4 $$\left\langle u^{2}\right\rangle =\left\langle r^{2}\left(\epsilon \right)\right\rangle =\left\langle {\left|r_{i}\left(\epsilon \right)-r_{i}\left(0\right)\right|}^{2}\right\rangle$$

Here, *r*_*i*_(ϵ) is the position of the *i*-th monomer in deformation ϵ, and *r* ^2^(ϵ) is obtained from the average of all the monomers. Experimentally, ⟨*u* ^2^⟩ can be measured via X-ray and neutron-scattering techniques.^59^ In MD simulations, ⟨*u* ^2^⟩ could be obtained from calculations of the mean-squared displacement (MSD) *r* ^2^(ϵ) of the center of mass of the monomers. This was measured from the sample through numerous global strain values to determine the local stiffness dependence on space and time, to be quantitative. The evolution of the molecular dynamic stiffness, as a function of the global deformation ϵ, is shown in Figure 4. Three distinct regimes can be identified: elastic regime (ϵ < 0.1) where the molecular stiffness remains constant when increasing strain, indicating a dominantly elastic response where dynamic mobility is weakly affected by deformation, that is in good agreement with the results of orientation dynamics of chain segments shown in Figure 3. Then in the strain-softening regime (0.1 < ϵ < 0.6) following the elastic region, the molecular stiffness gradually decreases with strain. This decrease reflects an increase in local dynamic mobility, associated with the development of plastic regions and structural rearrangements. Finally, the strain-hardening regime (0.6 < ϵ) follows in which the stiffness stabilizes at a low but nonzero value. This behavior suggests that, while the material undergoes significant plastic flow, molecular mobility becomes slightly suppressed due to the alignment and stretching of chains. This analysis confirms that local dynamic stiffness is sensitive to the evolving mechanical response of the material during deformation, providing microscopic insight into the transition from elastic to strain-softening and strain-hardening regimes.

**Figure 4:**
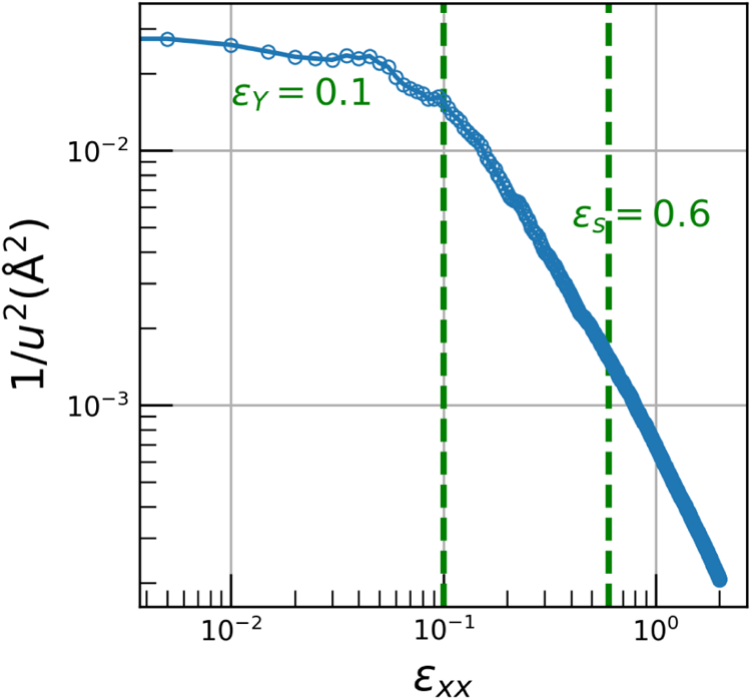
Evaluation of molecular stiffness via the Debye–Waller factor ⟨*u* ^2^⟩ during deformation.

### 3.4 Distribution of Local Strain Field during Deformation

The analysis presented in the previous subsections concerns the overall mechanical behavior of the glassy model PEO systems. Next, we investigate the mechanical heterogeneities by directly probing the distribution of the local strain field that is computed through a strain-per-atom formalism.^12^ Figure 5 depicts the probability distribution function and the standard deviation of the local strain, P­(ϵ) (inset and main plot of Figure 5a, respectively), as well as the probability distribution function and the standard deviation of the local deformation gradient *P*(*K*) (inset and main plot of Figure 5b, respectively). Data for different values of the applied global strain, ϵ_G_, are presented to illustrate the distributions at strain regions that include elastic-to-plastic and strain-softening to strain-hardening regimes. We observe that the distributions for the local strain and the gradient of deformation are symmetric (resembling Gaussian distributions), starting off as narrow peaks, indicating that most atoms reproduce the applied global strain and become progressively broader at higher strains, thus marking a more heterogeneous strain field at a local level within the polymer as strain increases.

**Figure 5:**
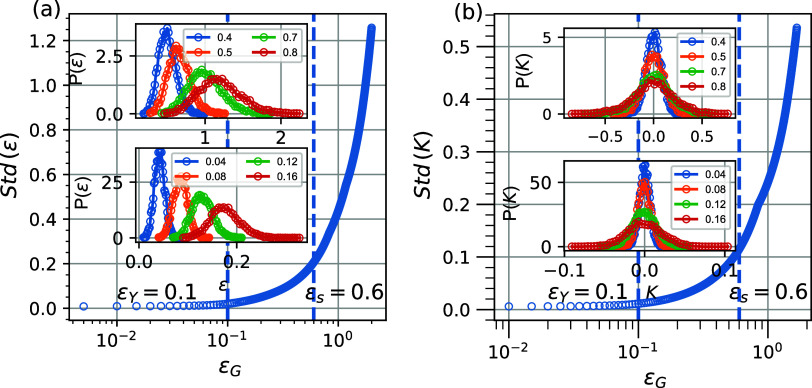
Standard deviation of local strain (a) and gradient of deformation (b) after fitting the probability distribution with the Gaussian distribution. (Inset) Probability distribution of local deformation (a) and deformation gradient (b) during the transition from elastic to plastic behavior, and the probability distribution of local strain during the transition from the softening to the hardening regime.

The standard deviation of *P*(ϵ) data reflects the strong variations and fluctuations associated with the heterogeneities in the distribution of local strain, and we highlight the onsets of different trends observed at the transition points of 0.1 and 0.6, in remarkable agreement with the previous markers of the elastic-to-plastic and strain-softening-to-strain-hardening transitions discussed above. Similarly, the distributions of the gradient of deformation shown in Figure 5b allow us to conclude that the strain is nonhomogeneous and becomes increasingly localized as heterogeneities emerge and become pronounced as we transition into the different realms of mechanical behavior.

### 3.5 Coupling between Local Density–Strain Heterogeneities

The indications mentioned above of strong changes in the nanostructure led us to further investigate the potential coupling between variations in the local density and the distributions of stress–strain fields. For this, we perform a 3D domain decomposition in the simulation domain, into small cubic boxes of length 5Å, and compute the average density within each box for a given applied global deformation. Figure 6 depicts the probability distribution function (PDF) of density, *P*(ρ), for a wide range of global strain values (inset), the average local density (red line), and its standard deviation (blue line) as a function of the applied deformation. As the applied global strain increases, both the average local density and the standard deviation of the local density manifest transitions between different behaviors, which also coincide with the critical values observed using the strain value markers discussed above. The average local density transitions from a very slow linear decrease in the elastic regime to a significant monotonic decrease in the strain-softening regime, in accordance with the rapid decrease in the volumetric strain (see Figure 2b), and an even more profound decreasing trend in the strain-hardening regime; again in agreement with the volumetric strain increase in that regime.

**Figure 6:**
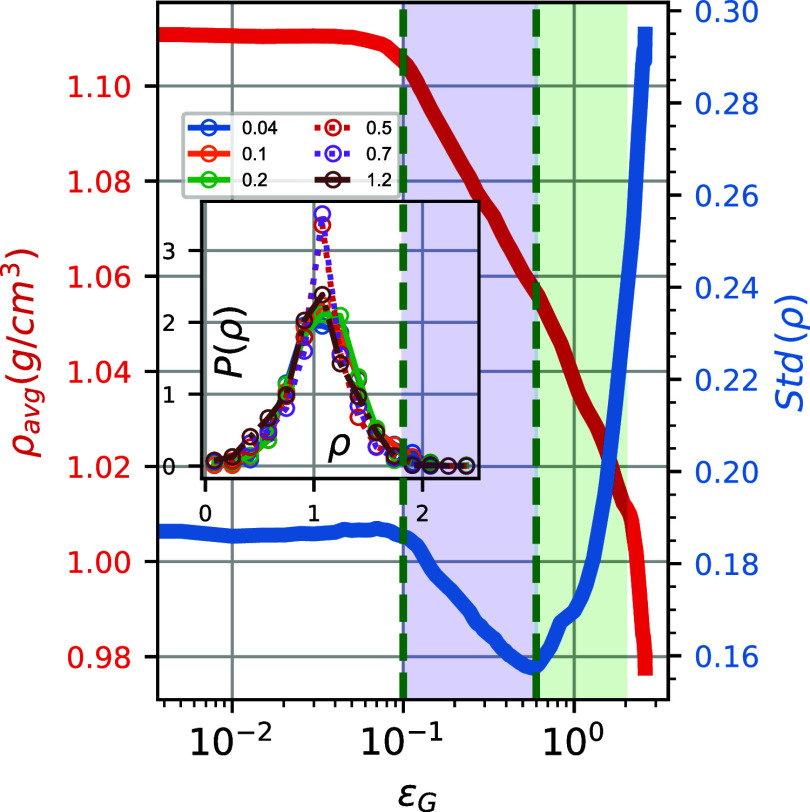
Evolution of the average local density, ρ_avg_, (red line, left axis) and the standard deviation of the local density, std­(ρ), (blue line, right axis), as a function of the applied deformation. (Inset) PDF of the local density for different applied strains.

The density heterogeneities are reflected in the standard deviation of the local density, shown in Figure 6. It is clear that in the elastic regime, the latter is almost constant, indicating that the density distribution is practically very similar to that of the equilibrium (undeformed) systems. Then, remarkably, the standard deviation of the local density decreases in the strain-softening regime, followed by a sudden transition to a strongly increasing behavior; i.e., widening of *P*(ρ) in the strain-hardening regime. We attribute this counterintuitive narrowing of *P*(ρ) during strain softening to the subtle effect of the decrease of the nonbonded energy (see Figure 2) in which polymer atoms (parts of vitreous segments) reorganize their relevant positions to minimize nonbonded interactions.

We examine the correlation among local strain, stress, and density, as shown in Figure 7, along with heat maps representing their spatial distribution in selected global strain values (elastic, strain-softening, and strain-hardening regimes). The results indicate that low-density regions correlate with high local strains in the elastic and strain-softening regimes (ϵ_G_ = 0.05). At the same time, Figure 7 does not reveal any clear correlation between the local stress and density.

**Figure 7:**
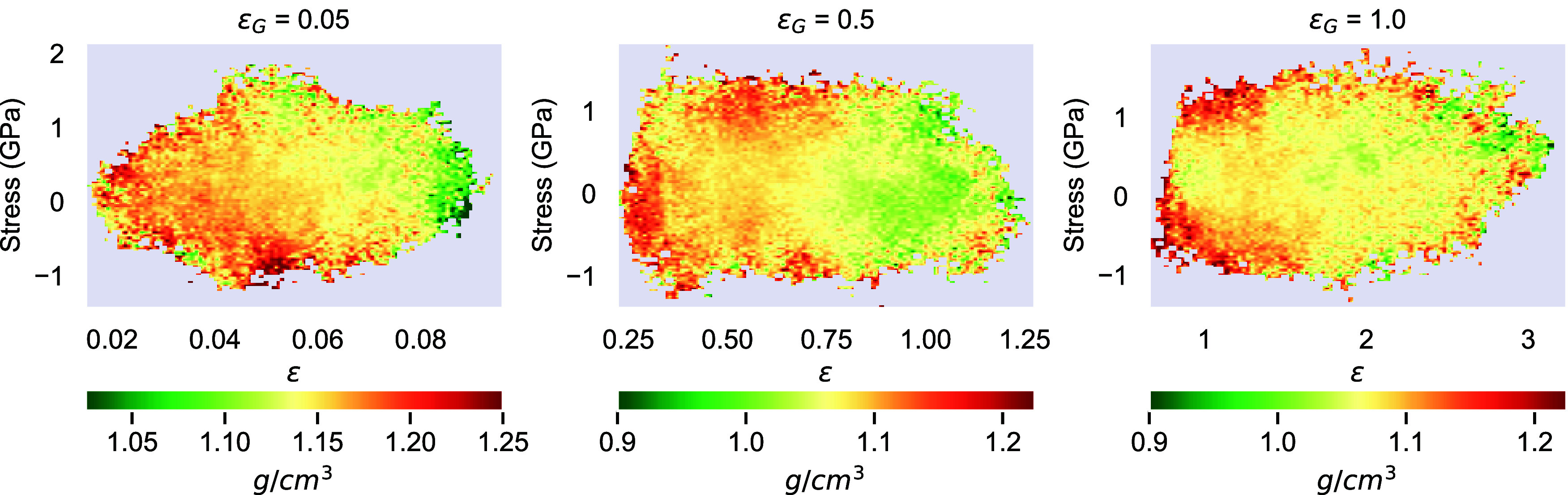
Correlation between local strain–stress–density for three applied global deformation ϵ_G_ fields in the elastic, strain-softening, and strain-hardening regimes. Heat maps are computed by performing a 3D domain decomposition in the simulation domain into small cubic boxes of 1 nm and computing the average local strain, local stress, and local density within each box. The various local density values are indicated by different colors.

In Figure 8, we present the Pearson correlation between local strain–density, stress–density, and chain orientation–density as a function of global deformation ϵ_G_. Starting at zero value due to the uniform nature of the strain and density profiles, once the applied global strain increases, the correlation between values of local density and local strain becomes weakly negative, increasing in magnitude, and reaching its maximum absolute value of 0.35, interestingly, within the strain-softening regime. After this, it begins to weaken as we move into the strain-hardening regime (Figure 8).

**Figure 8:**
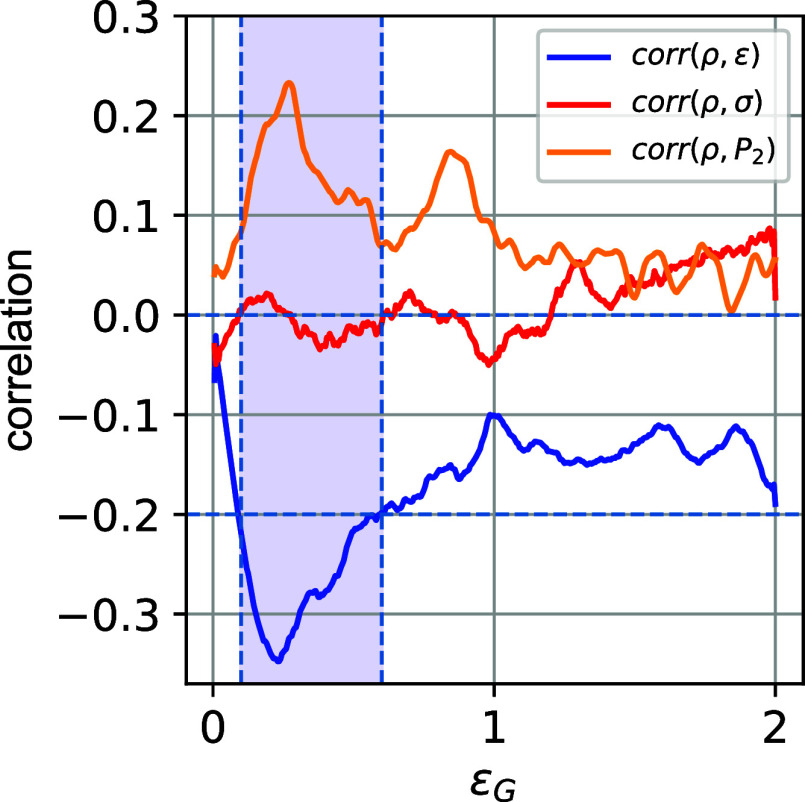
Pearson correlation between local strain–density, stress–density, and chain orientation (one Kuhn length *l*_k_, which is around 1 nm), and *P*_2_ density for a given applied global deformation ϵ_G_. The colored region presents the strain-softening regime.

Furthermore, a remarkable correlation between strain–density and *P*_2_–density values is observed in the elastic-to-plastic transition regime, that is shown in Figure 8. As strain increases, this cannot be sustained, and the standard deviation of *P*(ρ) increases dramatically, signifying the increased heterogeneity of the material in the strain-hardening regime, and the correlation becomes weaker. Our hypothesis is that the narrowing of *P*(ρ) at the elastic–plastic transition (see also Figure 6) is also related to the narrowing of the distribution of relaxation times (DRF) in the strain-softening regime and its broadening during strain hardening.^60^

Moreover, in terms of chain orientation, from the data shown in Figure 8, a moderate positive correlation of around 0.25 is found between *P*_2_ and the density in the strain-softening regime. This regime is characterized by the alignment and reorientation of the polymer chains, which affects the heterogeneities of the material. We should note that in previous works, using coarse-grained bead-spring models, no correlation was found between nonaffine displacements and the relevant local plastic deformation for glassy polymers.^61,62^ This further emphasizes the importance of accurately describing the chemical structure in the subnanometer, or sub-Kuhn, scale to investigate the potential correlations between atomic Virial stress and the local environment (density).

The discussion above emphasizes the important role of microstructure and density heterogeneities in mechanical behavior during the elastic–plastic transition. To further examine such a coupling, we analyze the local density data in low-, medium-, and high-density populations, defined via the probability distribution function of density *P*(ρ); that is, averaging over all boxes belonging to each class as shown in the inset of Figure 9a. In Figure 9a, we compute the local strain profiles as a function of global deformation for each of the low-, medium-, and average-density populations (i.e., averaging over all boxes belonging to each class). The strain curve of the high-density region follows global deformation in the elastic regime. Consequently, an affine deformation is expected in a region of high density, whereas nonaffine deformation is expected in regions of low and middle density in the elastic regimes. Generally, during tensile deformation, the regions of low density exhibit higher strain, although high values of the stress–strain diagram are observed.

**Figure 9:**
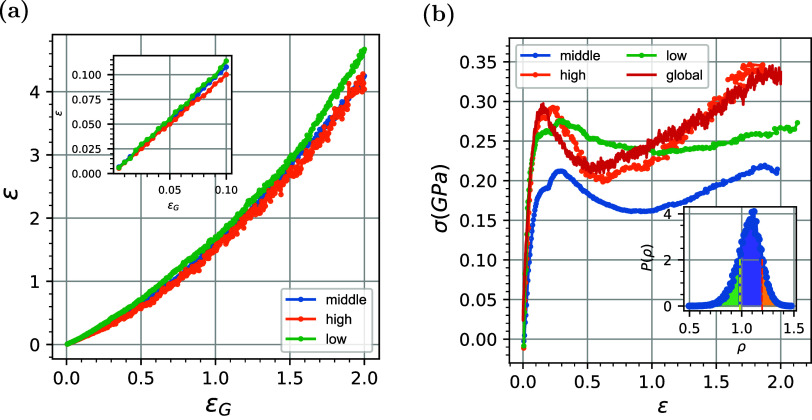
(a) Evolution of local average strain ϵ for three different regions with low-, middle-, and high-density values. (Inset) Evolution of local strain in the elastic region. (b) Stress–strain profiles in low-, medium-, and high-density populations.

Data on stress–strain profiles for each density population are shown in Figure 9b. It is clear that the stress–strain data curve of the entire material follows that of the high-density regions, indicating that the stress is mainly sustained in these regions, which are indeed expected to provide most of the rigidity of the material. Interestingly enough, the low-density regions follow the global stress only in the elastic regime; however, once they enter the plastic regime, they are able to dissipate energy and sustain a reduced stress, remaining longer in the strain-softening regime and even exhibiting a plateau-like behavior, with a much delayed entry into the strain-hardening regime compared to the rest of the material. The plateau region can be associated with energy/stress dissipation and enhanced mobility, hinting at an effectively dynamic, viscoelastic-like behavior of the low-density regions of the glassy systems. This finding aligns well with the results presented by the authors in^63,64^ where they show that supercooled liquids exhibit long-lived, long-range strain correlations expected only in solids.

In Figure 10, we compute the local chain orientation of segments with size one Kuhn length (with respect their equilibrium sate, *P*_2_, and with resepct the axis of deformation, *P_2_^xx^*) as a function of global deformation for each of the low-, medium-, and average-density populations. For *P*_2_, the chain orientation for each region is very close to each other and shows similar behavior as the global chain orientation presented in Figure 3. Concerning *P*_2_^*xx*^ its values in the low-density region are typically higher than in the regions of medium and high density, due to the additional free volume and the ability of the chain to reorient with respect to the axis of deformation *x*.

**Figure 10:**
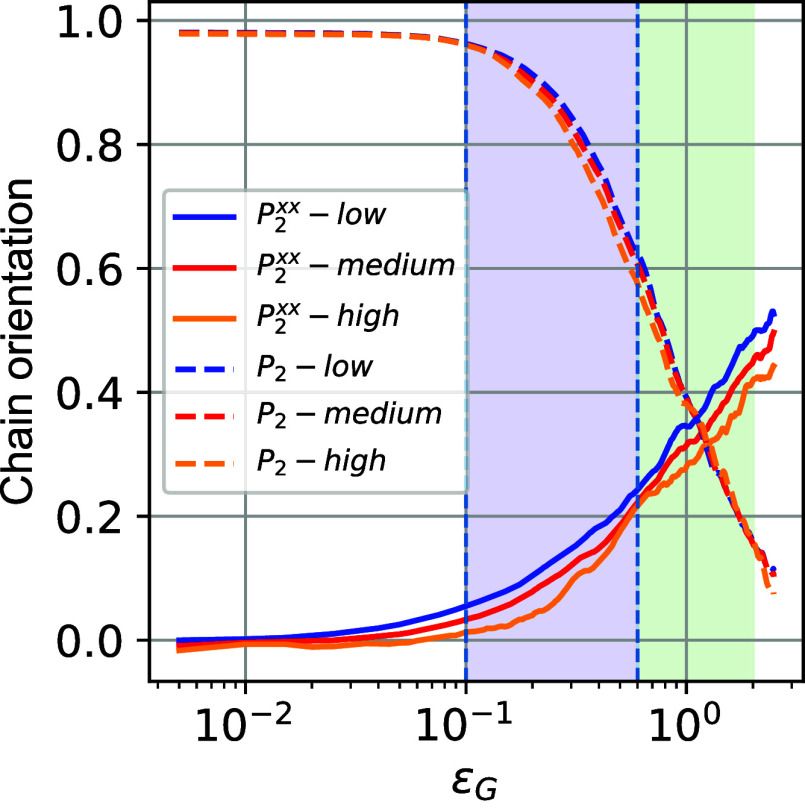
Evolution of chain orientation of a vector of one Kuhn length with respect its equilibrium state,*P*_2_, and with respect the axis of deformation,*P*_2_^*xx*^, computed as averages for three different populations of low, middle, and high density.

## 4 Conclusions

In this study, we explored the atomistic and microstructural underpinnings of the mechanical response of polymers undergoing imposed tensile strain and transitioning from elastic to the plastic regimes of strain softening and strain hardening. Through detailed atomistic molecular dynamics simulations, our investigation has shed light on the critical role of microstructure and heterogeneity in homogeneous systems of glassy, unentangled polymers.

Probing the complex interplay of atomistic and chain-level phenomena has unraveled various markers across different levels of description, signifying transitions between the different regimes that are manifested on the macroscopic/engineering scale. The emergence of an inflection point in the local versus global strain diagram and deviations in local density serve as key indicators of the transition, providing new insights into the atomistic mechanisms underpinning the mechanical response of polymers. The various markers that have emerged include deviations in average local strain resulting in transition points in the local versus global strain diagram, variations in the bonded and nonbonded stress, reorientation of polymer chains, strain localization through deformation gradient analysis, and heterogeneities in local strain and density. These indicators are in remarkable agreement, paving the way for a deeper understanding of material behavior at the microscopic level.

Our results indicate that during deformation in glassy polymeric systems undergoing tensile tests, local heterogeneities in density and local strain become prominent for strain values beyond the linear regime. At the local level, regions of lower density (increased free volume) appear that activate the reorganization of vitreous segments, thus reducing the nonbonded (mainly van der Waals type) stresses. At the same time, such regions exhibit higher local strain values, resulting in an average local strain that is larger than that of the imposed global deformation. Entering the plastic regime, these lower density regions are able to dissipate energy and sustain a reduced stress, hinting at increased mobility and effective viscoelastic-like dynamic behavior, before finally entering the strain-hardening regime at much larger strain values compared to the rest of the material. The regions of higher local density, on the other hand, enter the strain-hardening regime at earlier strain values and, in general, provide most of the stress response of the material.

The correlation of plastic behavior with local density heterogeneities, illustrated by our study, opens new exciting questions on microstructural, atomistic origins of plasticity as well as on the evolution of the heterogeneities as part of the dynamic response and relaxation behavior of glassy polymeric systems. An interesting question is how the distribution of relaxation times is affected in the plastic regime due to the coupling of local density–strain heterogeneities. We hope that our study will stimulate more work toward the development of predictive models that can simulate material behavior under diverse mechanical loads. Moreover, extending these methods to a broader range of polymer systems, including nanocomposites and biobased polymers, could facilitate the design of next-generation materials with tailored mechanical properties.
